# Ultra-Processed Food Consumption and Risk of Overweight or Obesity in Chinese Adults: Chinese Food Consumption Survey 2017–2020

**DOI:** 10.3390/nu15184005

**Published:** 2023-09-16

**Authors:** Feng Pan, Tongwei Zhang, Weifeng Mao, Fanglei Zhao, Dechun Luan, Jianwen Li

**Affiliations:** 1 China National Center for Food Safety Risk Assessment, Beijing 100022, China; panfeng@cfsa.net.cn (F.P.); zhangtongwei@cfsa.net.cn (T.Z.); maoweifeng@cfsa.net.cn (W.M.); zhaofanglei@cfsa.net.cn (F.Z.); 2 Liaoning Center for Disease Control and Prevention, Shenyang 110005, China; dechunluan@163.com

**Keywords:** ultra-processed foods, consumption, overweight, obesity, adults, Chinese Food Consumption Survey

## Abstract

Overweight and obesity have increased rapidly in the past few decades in China, and less research has focused on the association between the consumption of ultra-processed foods (UPFs) and overweight or obesity in Chinese adults. The objective of this study was to determine the relationship of UPF consumption with the risk of overweight or obesity in Chinese adults. Residents aged 18 years and above who participated in the nationally representative survey Chinese Food Consumption Survey in 2017–2020 were included in this study. Dietary intake data were collected via non-consecutive three-day 24 h dietary recalls and weighing household foods and condiments. According to the NOVA food classification system, UPFs were classified depending on the purpose and extent of food processing. Multiple logistic regression and multivariate-adjusted restricted cubic spline (RCS) regressions were performed to estimate the association between UPF consumption (categorized into quartiles: quartile 1 (Q1), quartile 2 (Q2), quartile 3 (Q3), and quartile 4 (Q4)) and risk of overweight or obesity. A total of 38,658 adults aged 18 years and above participated in the present study. The prevalence of overweight and obesity in adults was 33.0% (95% CI: 32.91–33.10) and 9.6% (95% CI 9.55–9.67), respectively. After a multivariable adjustment, the risk of overweight or obesity was increased by 10% in Q3 (OR: 1.10, 95% CI: 1.04–1.17) compared to Q1 as a reference. Women (OR: 1.10, 95% CI: 1.01–1.20) and adults living in small and medium-sized cities (OR: 1.13, 95% CI: 1.01–1.26) with higher UPF consumption had higher odds of overweight or obesity. Nevertheless, the effect of higher UPF consumption on the risk of overweight or obesity was relatively weak for overall adults in China. The top three categories of subgroups consumption of UPFs were 1: sugar-sweetened beverages; 2: sauces, cheeses, spreads, and gravies; and 3: ultra-processed breads and breakfast cereals. These findings provide evidence that higher UPF consumption was positively associated with overweight or obesity, which was defined based on Chinese criteria among women and adults living in small and medium-sized cities in China. Further studies, such as intervention trials, are needed to identify the mechanism of correlation between the consumption of UPFs and health-related outcomes in Chinese adults. From a public health perspective, with the prevalence of overweight and obesity growing and the increase in UPF consumption in Chinese adults, it is necessary to promote healthy food intake and a balanced diet through active nutritional education actions for overweight and obesity prevention and control.

## 1. Introduction

Overweight and obesity are defined as abnormal or excessive fat accumulation, which may impair health. With the rapid development of the global economy and social progress, the prevalence of overweight and obesity has risen significantly over the past few decades [1,2]. In 2015, a total of 603.7 million adults worldwide were obese [3]. In 2021, WHO data show that more than 1.9 billion adults (39% of the world’s adult population) aged 18 years and above were overweight in 2016 [4]. With social and economic changes and lifestyle shifts, a similar situation has emerged in China. Findings of the Report on Chinese Residents’ Chronic Diseases and Nutrition 2020 showed that more than half of adults were overweight or obese in China [5]. Nowadays, China has the highest number of people with overweight and obesity in the world [6]. Overweight and obesity are important risk factors for cardiovascular and cerebrovascular diseases, diabetes, renal disease, and various cancers, and they also increase the risk of noncommunicable diseases, which are the leading causes of death [7,8,9]. Furthermore, increasing rates of overweight and obesity place huge economic and medical burdens on society [6].

Diet, as a determinant for overweight and obesity, plays a key role in the development of overweight and obesity. China is facing a nutrition transition from the traditional Chinese diet to the westernization of dietary patterns [10]. The Chinese are consuming more packaged processed foods, cakes, cookies, and pastries, while the consumption of vegetables and fruits has decreased over the past few decades [11]. Excessive total calories from foods such as sugar-sweetened beverages (SSBs) and processed foods with low nutritional value and high energy density are more likely to cause overweight and obesity [12].

Ultra-processed foods are commonly high energy dense and contain added sugar, saturated fat, and salt, but they are low in fiber and micronutrients. Based on the degree of food processing, all foods and food products are classified by the NOVA food classification system, which is a new classification considering the nature and purpose of industrial processing [13]. NOVA classifies all foods and food products into four groups: Group 1: unprocessed or minimally processed foods (MPFs), Group 2: processed culinary ingredients (PCIs), Group 3: processed foods (PFs), and Group 4: ultra-processed foods (UPFs) [14]. UPFs are extremely convenient, highly palatable, and profitable foods made from inexpensive ingredients and refined extracts from foods using a series of industrial formulations and processes [13]. There is a growing body of research on the relationship between higher UPF consumption and human health with the increasing consumption of UPFs [15,16,17]. Consistent evidence has linked higher UPF consumption to overweight and obesity in multiple systematic reviews and meta-analyses [18,19,20,21]. The cross-sectional National Health and Nutrition Examination Survey (NHANES 2005–2014) found that compared to the lowest consumption of UPFs (0–36.5% of energy), adults (aged 20–64 years) with the highest consumption of UPFs (74.2–100% of energy) was associated with 48% and 53% higher odds of overweight (BMI ≥ 25 kg/m^2^; OR: 1.48, 95% CI: 1.25, 1.76) and obesity (BMI ≥ 30 kg/m^2^; OR: 1.53, 95% CI: 1.29, 1.81), respectively [22]. The Brazilian longitudinal study of adult health (ELSA-Brasil) showed that the risk of incident overweight or obesity increased by 20% (RR: 1.20, 95% CI: 1.03, 1.40) among adults with the fourth consumption of UPFs quartile compared to the first quartile of UPFs consumption [23]. With regards to Chinese adults, Li et al. showed that the mean daily UPF consumption increased by close to four times from 1997 to 2011, and higher UPF consumption was associated with an increased risk of overweight/obesity (BMI ≥ 25 kg/m^2^; OR: 1.45, 95% CI: 1.21, 1.74), according to the data of China Health and Nutrition Survey (CHNS 1997–2011) [24]. However, few studies have focused on the association between UPF consumption and overweight or obesity among Chinese adults in recent years, especially when overweight and obesity were defined based on Chinese criteria.

In view of the increasing prevalence of overweight and obesity and higher UPF consumption in Chinese adults, it is necessary to clarify the correlation between UPF consumption and overweight or obesity so as to put forward measures to prevent overweight and obesity through diet. We performed the present study to explore the association between UPF consumption and overweight or obesity in Chinese adults using nationally representative survey data from the Chinese Food Consumption Survey in 2017–2020 (CFCS 2017–2020). The objective of this study was to come up with a more accurate estimation of the association between UPF consumption and overweight or obesity and provide targeted suggestions and measures for preventing and controlling overweight and obesity through dietary behavior in Chinese adults.

## 2. Materials and Methods

### 2.1. Study Design and Population

The study data were obtained from the Chinese Food Consumption Survey in 2017–2020 (CFCS 2017–2020). The CFCS is a nationally representative cross-sectional study conducted by China National Center for Food Safety Risk Assessment to examine the food consumption status and assess food, nutrient intakes, and food safety risk in the Chinese population. The CFCS was conducted at 126 investigated sites across 23 provinces in 2017–2020. This survey used a multistage stratified cluster random sampling method to collect the sample information, including food consumption and diet, food processing methods, food contact materials, demographic geography, economic development, and physical examination.

**Figure 1:**
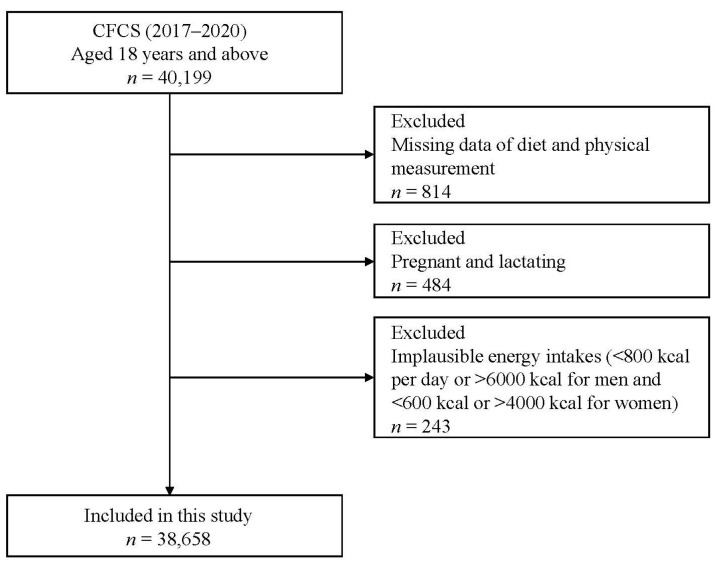
Flowchart of the selection process for eligible participants in this study.

We excluded the subjects according to the following exclusion criteria: (1) participants with deficiency of dietary and physical measurement data, 814 in total; (2) pregnant or lactating women, 484 in total; and (3) participants with implausible energy intakes (<800 kcal/day or >6000 kcal/day for men; <600 kcal/day or >4000 kcal/day for women), 243 in total. Finally, a total of 38,658 adults aged 18 years or older were included in the present study (Figure 1). The CFCS was approved and supported by the National Health Commission of the People’s Republic of China for food safety risk assessment in accordance with the Food Safety Law of the People’s Republic of China. Therefore, the current study was waived from ethical review. Written informed consent was obtained from all subjects.

### 2.2. Definition of UPFs and Dietary Assessment

According to the NOVA food classification system, food items were categorized into four groups, including MPFs, PCIs, PFs, and UPFs [11]. UPFs included products like packaged snacks, sweets, sugar-sweetened beverages (SSBs), ice cream, energy bars, mass-produced packaged breads, pizza dishes, pre-prepared pies, bacon, and sausages that have undergone intensive industrial processing, such as pre-frying, molding, extrusion, and hydrogenation. For food items that could not be clearly classified, one or more food substances present in the ingredient list that are not used in kitchens, such as fructose, inverted sugar, maltodextrin, hydrolyzed proteins, mechanically separated meat, and hydrogenated oil, were identified as UPFs.

We used non-consecutive three-day 24 h dietary recalls (including one weekend and two weekdays) for all individuals to collect dietary data. Two 24 h dietary recall days were separated by at least three days. The first and third 24 h dietary recall days were separated by at least nine days. For the consumption of oil and various seasonings, trained investigators weighed food items in the household inventory. Total energy and nutrient intake per day were calculated using the Chinese Food Composition Table [25,26].

### 2.3. Definition of Overweight and Obesity

Anthropometric data were collected using standardized protocol by trained health workers with strict quality control during the household investigation. The measurements of weight and height were carried out in accordance with the requirements of WS/T 424-2013 “Anthropometric measurements method in health surveillance.” Weight was measured in kilograms (kg) without shoes and in light clothing to the nearest 0.1 kg using an electronic weight scale (TC-200k). Height was measured in centimeters (cm) without shoes to the nearest 0.1 cm using a portable stadiometer.

Overweight or obesity was defined based on Chinese criteria [27]. Body mass index (BMI) was calculated as body weight (kg) divided by the square of height (m^2^). Overweight was defined as 24.0 kg/m^2^ ≤ BMI < 28.0 kg/m^2^, and obesity was defined as BMI ≥ 28 kg/m^2^ in adults.

### 2.4. Covariates

In this study, multiple covariates were involved, including gender, age, education level, individual annual income, occupation, alcohol drinking status, labor intensity, geographical location, total energy intake, protein, fat, carbohydrate, dietary vitamin A, dietary vitamin C, dietary calcium, and dietary sodium. Age was divided into three groups (18–44 years, 45–59 years, and 60 years and above). Education level was divided into three groups (primary school or below, secondary school, and college or above). Individual annual income was divided into five groups (CNY <10,000, 10,000–39,999, 40,000–99,999, >100,000, and no response). Occupation was separated into six groups (student, retired or unemployed, professionals, business service, manual labor, and others). Alcohol drinking status over the past month was divided into two groups (yes and no). Labor intensity was divided into three groups (light, medium, and heavy). Residence was separated into three groups (metropolises, small and medium-sized cities, and rural areas). The regions were divided into the eastern (Beijing, Tianjin, Hebei, Liaoning, Shanghai, Jiangsu, Zhejiang, Fujian, Shandong, and Guangdong), central (Inner Mongolia, Jilin, Heilongjiang, Jiangxi, Henan, Hubei, Hunan), and western (Guangxi, Chongqing, Guizhou, Yunnan, Shaanxi, and Gansu).

### 2.5. Statistical Analysis

Categorical variables were described by n, percentage (%), and compared using the χ^2^ test. Continuous variables were described using mean and standard deviation and compared using the Kruskal–Wallis test. Multiple logistic regression analysis was used to quantify the association between UPF consumption (categorized into quartile: Q1, Q2, Q3, and Q4) and the risk of overweight or obesity. We used the median value of each quartile of UPF consumption as a continuous variable to test the linear trends between UPF consumption and the risk of overweight or obesity. For the sensitivity analyses, we performed a stratified analysis using multiple covariates and an interaction analysis to estimate the effect of stratification factors on the association between UPF consumption and the risk of overweight or obesity. To explore the nonlinear relationship between UPF consumption and ORs of overweight or obesity, multivariate-adjusted restricted cubic spline (RCS) regressions were performed in the primary analysis. Statistical analyses were conducted using SAS 9.4 (SAS Institute, Inc., Cary, NC, USA), and the plot was generated in R (version 4.1.0). All statistical tests were two-tailed and considered significant at *p* < 0.05.

## 3. Results

### 3.1. Baseline Characteristics

As shown in Table 1, a total of 38,658 adults aged 18 years and above participated in the present study. Participants with higher UPF consumptions were more likely to have higher education levels and light labor intensity, live in metropolis, and have higher BMI, higher energy intake, higher protein intake, higher fat intake, higher carbohydrate intake, higher vitamin A intake, higher vitamin C intake, higher calcium intake, and higher sodium intake compared with those in the lowest quartile of UPF consumption (*p* < 0.01). Among the highest quartile of UPF consumption groups, more adults were 18–44 years old with college or above education levels and CNY 40,000–99,999 individual annual income and worked in technical or business services (*p* < 0.001).

### 3.2. Associations of UPF Consumption with Overweight or Obesity

Table 2 explores the associations of UPF consumption with overweight or obesity. Between 2017 and 2020, the prevalence of overweight and obesity in adults was 33.0% (95% CI: 32.91–33.10) and 9.6% (95% CI 9.55–9.67), respectively.

After the adjustment for confounding factors, including gender, age, education level, individual annual income, occupation, drinking past month, labor intensity, and place and region of residence (Model 2), the risk of overweight or obesity was increased by 8% in the highest quartile (Q4) with UPF consumption (OR: 1.08, 95% CI: 1.02–1.14, *p* trend: 0.690), with the lowest quartile (Q1) as a reference. With further adjustment based on total energy, protein, fat, carbohydrate, vitamin A, vitamin C, calcium, and sodium intake (Model 3), only quartile 2 (Q2) and quartile 3 (Q3) had increased risk of overweight or obesity (OR: 1.14, 95% CI: 1.07–1.20; OR: 1.10, 95% CI: 1.04–1.17) compared to Q1. The risk of overweight or obesity was increased by 3% in the highest quartile (Q4) of UPF consumption (OR: 1.03, 95% CI: 0.97–1.09, *p* trend: 0.241) with no statistical significance.

For the associations of UPF consumption with overweight, after adjusting for covariates (Model 2), the risk of overweight was increased by 7% (OR: 1.07, 95% CI: 1.01–1.15, *p* trend: 0.836). For Model 3, no correlation was observed between the highest quartile (Q4) UPF consumption and overweight (OR: 1.03, 95% CI: 0.97–1.10, *p* trend: 0.236) compared with the lowest quartile (Q1) consumption. For the associations of UPF consumption with obesity, compared to Q1, no correlation was observed between the highest quartile (Q4) UPF consumption and obesity (OR: 1.02, 95% CI: 0.92–1.14, *p* trend: 0.556).

### 3.3. Stratified Analyses of Overweight or Obesity and UPFs Consumption

Table 3 presents the sensitivity analyses of overweight or obesity risk and UPF consumption. The results showed that the positive association of UPF consumption with the risk of overweight or obesity was consistent in women and subjects living in small and medium-sized cities with Q4. In addition, gender and place of residence had an interactive effect on the association between UPF consumption and risk of overweight or obesity (*p* < 0.05).

### 3.4. Nonlinear Relationship between UPF Consumption and ORs of Overweight or Obesity

**Figure 2:**
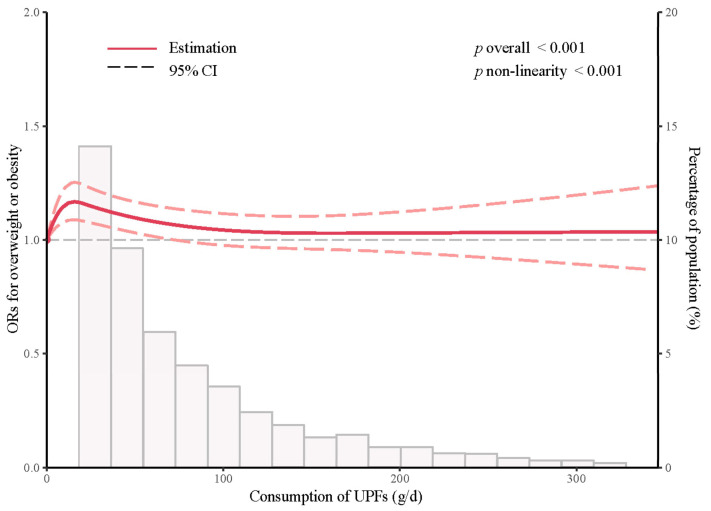
Association of UPF consumption and ORs of overweight or obesity. Red solid line shows ORs estimated after being adjusted for gender, age, education level, individual annual income, occupation, alcohol drinking status, labor intensity, geographical location, total energy intake, dietary protein, fat, carbohydrate, vitamin A, vitamin C, calcium, and sodium; pink-dashed lines indicate 95% CIs.

We used RCS to model and visualize the relationship between UPF consumption and ORs of overweight or obesity (Figure 2). The plot shows an inverted U-shaped relationship within the lower range of UPF consumption, and then the risk of overweight or obesity is relatively flat about 100 g/d above thereafter (*p* non-linearity < 0.001). Furthermore, there is no clear linear trend between the risk of UPF consumption and overweight or obesity.

### 3.5. Subgroups Consumption of UPFs among Overweight or Obesity Groups

**Figure 3:**
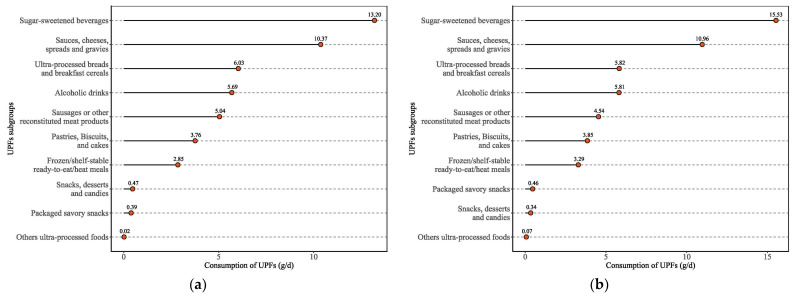
Subgroups’ consumption of UPFs among overweight or obesity groups. (**a**) Overweight group; (**b**) obesity group.

Figure 3 shows the subgroups’ consumption of UPFs in overweight or obesity groups. The top three categories of subgroups’ consumption of UPFs were sugar-sweetened beverages (13.20 g/d among overweight group, 15.53 g/d among obesity group); sauces, cheeses, spreads, and gravies (10.37 g/d among overweight group, 10.96 g/d among obesity group); and ultra-processed breads and breakfast cereals (6.03 g/d among overweight group, 5.82 g/d among obesity group). The lowest three categories of subgroups’ consumption of ultra-processed foods were snacks, desserts, and candies; packaged savory snacks; and other ultra-processed foods among the overweight group and packaged savory snacks; snacks, desserts, and candies; and others ultra-processed foods among the obesity group.

## 4. Discussion

The findings of this study demonstrate the association between UPF consumption and overweight or obesity among Chinese adults aged 18 years and above through nationwide population-based data from CFCS from 2017–2020. As the consumption of UPFs increased, higher UPF consumption was found to be positively correlated with overweight or obesity among women and adults living in small and medium-sized cities after adjusting for potential confounders. The highest UPF consumptions were associated with 10% and 13% significant increases in the risk of overweight or obesity in women and adults living in small and medium-sized cities, respectively. Nevertheless, the effect of higher UPF consumption on the risk of overweight or obesity was relatively weak for overall adults. The top three categories of subgroups’ consumption of UPFs were 1: sugar-sweetened beverages; 2: sauces, cheeses, spreads, and gravies; and 3: ultra-processed breads and breakfast cereals.

Multiple similar cross-sectional studies have been conducted in several countries, including the UK [28], Brazil [29], Australia [30], Canada [31], and France [32]. Individuals with higher UPF consumption had significantly higher odds of being overweight or obese to varying degrees. Furthermore, many prospective cohort studies have also found a causal association between higher UPF consumption and being overweight or obese. Mendonca, R. D., et al. showed that participants with the highest quartile of UPF consumption had a higher risk of developing overweight or obesity (HR: 1.26; 95% CI: 1.10, 1.45) than those with the lowest quartile of consumption in a prospective Spanish cohort [33]. A prospective cohort study of UK Biobank found that adults aged 40–69 years had a significantly higher risk of developing overall obesity (HR: 1.79; 95% CI: 1.06, 3.03), with the highest quartile of UPF consumption [34]. The European Prospective Investigation into Cancer and Nutrition (EPIC) study found that participants with the highest quintile of UPF consumption were associated with a 15% greater risk (95% CI: 1.11, 1.19) of becoming overweight or obese among adults from nine European countries. However, higher UPF consumption was associated with a negligible weight gain of 0.12 kg over 5 years [35]. For the Chinese population, UPF consumption is still at a relatively low level compared to Europe and the United States [21]. Therefore, the association between higher UPF consumption and overweight or obesity is relatively weak for adults.

Several potential mechanisms may explain the association between higher UPF consumption and overweight or obesity. Firstly, an obvious feature of UPFs is that UPFs have higher energy density compared to a traditional diet [36]. In addition, UPFs tend to have a higher content of saturated fat, added sugar, and salt, along with less fiber, micronutrients, and vitamins [14,37]. Therefore, as UPF consumption increases, energy intake increases at the same time, leading to poorer nutritional quality on average and increasing the incidence of various chronic conditions, especially overweight and obesity [38,39]. Secondly, the deep processing of food and food packaging materials can result in the production of potentially toxic compounds and contaminants, such as polycyclic aromatic hydrocarbons, furans, acrolein, urinary phthalate, heterocyclic amines, bisphenols, and phthalates [40,41]. Studies have shown that bisphenols are associated with higher risks of obesity and a variety of noncommunicable diseases [42,43,44]. Thirdly, UPFs may result in a disruption of food matrices during production and processing and affect the duration of chewing, transit time, and digestibility [45,46]. Moreover, the high palatability and wide accessibility of UPFs lead to increased eating rates and overconsumption and tend to increase the risk of overweight or obesity [21].

In the present study, we found that higher UPF consumption was more associated with women, not men, after adjusting for potential confounders. The observed gender-based differences in our study were consistent with the results of some previous studies. In a nationally representative cross-sectional study of Korean adults aged 19–64 years from the Korea National Health and Nutrition Examination Survey (KNHANES), 2016–2018, women with the highest consumption of UPFs had higher odds of being obese (BMI > 25 kg/m^2^; OR: 1.51, 95% CI: 1.14, 1.99), while no association was found in men [47]. Filippa Juul et al. showed that US adults aged 20–64 years were associated with 2.37 kg/m^2^ higher BMI among women (95% CI: 1.58, 3.17), but only 0.79 kg/m^2^ higher BMI among men (95% CI: 0.18, 1.39), according to NHANES 2005–2014 [22]. Another cross-sectional study in Brazil found that women with the highest consumption of UPFs had higher risk (OR: 1.69, 95% CI: 1.12, 2.54) for excess weight (overweight and obesity). However, higher UPF consumption had no relationship (OR: 1.17, 95% CI: 0.78, 1.76) with excess weight (overweight and obesity) in men [29]. Previous studies have offered some explanations for the gender differences. On the one hand, food choices differ between women and men. Women tended to consume a higher percentage of energy from UPFs including sweet snacks, cakes, cookies and pies, ice cream, and desserts. Therefore, women had more total energy and sugar intake than men [22]. On the other hand, women may be more affected in the biological metabolism and adiposity because of foods with high glycemic indexes (GIs) and glycemic loads [48,49]. As a consequence, higher consumption of UPFs, such as refined carbohydrates and sugary foods with high GIs, makes it easier to gain excess weight [22]. The specific metabolic mechanism and unmeasured confounders warrant the exploration of the reasons for gender differences.

We also found that the association was more pronounced among adults living in small and medium-sized cities compared to metropolis and rural areas. In the past two decades, society and the economy have developed rapidly, and the urbanicity rate has been on the rise in China [50]. Individuals can obtain higher income, educational levels, and variety of food with rapid urbanicity [51]. However, rapid urbanicity and rural-to-urban shift have impacts on the food choices, eating habits, levels of physical activity, and health condition of individuals [52]. Adults with higher educational and economic levels may have higher UPF consumption, as we found. Dietary fat intake increases with higher UPF consumption, which can also contribute to increased calorie intake and weight gain [53]. In addition, people are more likely to ignore nutritional factors when making food choices, while the development of cities is not balanced with the improvement of nutrition knowledge. As a result, the consumption of UPFs may increase, as well as being overweight or obese. Further research is needed to investigate linkages between urbanization, food consumption, and nutritional health, and adequate nutritional recommendations should be made.

To the best of our knowledge, this study is the first nationally representative cross-sectional study to demonstrate the association between UPF consumption and the risk of overweight or obesity defined based on Chinese criteria in Chinese adults. Of note, detailed dietary data were collected through previously validated weighing of foods and condiments in household inventories of non-consecutive three-day 24 h dietary recall, which could contain more accurate and various food types [54]. In addition, we used the updated NOVA food classification system and objective criteria to classify foods by their level of processing. Nevertheless, potential limitations should be considered. First, a causal association between UPF consumption and overweight or obesity cannot be drawn because of the cross-sectional study. Second, the possibility of residual confusion could not be completely avoided, though potential confounding factors were adjusted. Third, long-term diet habits could not be completely reflected by 24 h dietary recall, and the retrospective method might be subject to recall bias. Thus, more prospective cohort studies are needed to explore the correlation between UPF consumption and health outcomes with food frequency questionnaires based on 24 h dietary recall.

## 5. Conclusions

In conclusion, the present study found that higher UPF consumption was positively associated with overweight or obesity defined based on Chinese criteria among women and adults living in small and medium-sized cities. Nevertheless, the effect of higher UPF consumption on the risk of overweight or obesity was relatively weak for overall adults in China. Further studies, such as intervention trials and longitudinal studies, are needed to identify the mechanisms of correlation between consumption of UPFs and health-related outcomes in Chinese adults. From a public health perspective, with the prevalence of overweight and obesity growing and increased UPF consumption in Chinese residents, it is necessary to promote healthy food intake and a balanced diet through active nutritional education actions for overweight and obesity prevention and control.

## Figures and Tables

**Table 1:** Demographic and basic characteristics of participants by quartile of UPF consumption.

	Quartile of UPFs (g/day)				
	Q1 (<4.6)	Q2 (4.6–18.2)	Q3 (18.2–59.1)	Q4 (>59.1)	*p*-Value
Gender					<0.001
Men	4149 (42.9)	4427 (45.8)	4597 (47.6)	5394 (55.8)	
Women	5515 (57.1)	5238 (54.2)	5066 (52.4)	4272 (44.2)	
Age					<0.001
18–44	3786 (39.2)	3705 (38.3)	3891 (40.3)	4679 (48.4)	
45–59	3877 (40.1)	3837 (39.7)	3787 (39.2)	3407 (35.3)	
≥60	2001 (20.7)	2123 (22.0)	1985 (20.5)	1580 (16.4)	
Education level					<0.001
Primary school or below	2685 (27.8)	2200 (22.8)	1958 (20.3)	1549 (16.0)	
Secondary school	5319 (55.0)	5419 (56.1)	5300 (54.9)	5080 (52.6)	
College or above	1660 (17.2)	2046 (21.2)	2405 (24.9)	3037 (31.4)	
Individual annual income (CNY)					<0.001
<10,000	3009 (31.1)	2343 (24.2)	2161 (22.4)	2039 (21.1)	
10,000–39,999	5196 (53.8)	5461 (56.5)	5284 (54.7)	5148 (53.3)	
40,000–99,999	791 (8.2)	1133 (11.7)	1386 (14.3)	1534 (15.9)	
>100,000	213 (2.2)	276 (2.9)	345 (3.6)	358 (3.7)	
No response	455 (4.7)	452 (4.7)	487 (5.0)	587 (6.1)	
Occupation					<0.001
Student	123 (1.3)	110 (1.1)	126 (1.3)	244 (2.5)	
Retired or unemployed	3262 (33.8)	3299 (34.1)	2999 (31.0)	2463 (25.5)	
Professionals	1212 (12.5)	1422 (14.7)	1649 (17.1)	1876 (19.4)	
Business service	1436 (14.9)	1696 (17.6)	1890 (19.6)	2115 (21.9)	
Manual labor	2596 (26.9)	2218 (23.0)	2132 (22.1)	2024 (20.9)	
Others	1035 (10.7)	920 (9.5)	867 (9.0)	944 (9.8)	
Drinking past month					<0.001
Yes	2235 (23.1)	2620 (27.1)	3069 (31.8)	3931 (40.7)	
No	7429 (76.9)	7045 (72.9)	6594 (68.2)	5735 (59.3)	
Labor intensity					<0.001
Light	5316 (55.0)	5747 (59.5)	5980 (61.9)	5873 (60.8)	
Medium	3404 (35.2)	3275 (33.9)	3041 (31.5)	3136 (32.4)	
Heavy	944 (9.8)	643 (6.7)	642 (6.6)	657 (6.8)	
Place of residence					<0.001
Metropolis	2335 (24.2)	3085 (31.9)	3172 (32.8)	3504 (36.3)	
Small and medium-sized cities	3069 (31.8)	3040 (31.5)	3286 (34.0)	3083 (31.9)	
Rural areas	4260 (44.1)	3540 (36.6)	3205 (33.2)	3079 (31.9)	
Region of residence					<0.001
Eastern regions	3403 (35.2)	4958 (51.3)	4911 (50.8)	4987 (51.6)	
Central regions	3579 (37.0)	2466 (25.5)	2401 (24.9)	2801 (29.0)	
Western regions	2682 (27.8)	2241 (23.2)	2351 (24.3)	1878 (19.4)	
BMI (kg/m^2^)	23.4 ± 3.3	23.7 ± 3.3	23.7 ± 3.4	23.6 ± 3.4	<0.001
Energy (kcal/day)	1848.2 ± 595.3	1807.63 ± 590.4	1897.6 ± 579.6	2150.0 ± 663.3	<0.001
Protein (g/day)	62.1 ± 23.4	62.9 ± 23.9	66.1 ± 24.0	72.8 ± 26.1	<0.001
Fat (g/day)	65.4 ± 34.9	67.5 ± 35.1	74.6 ± 36.4	85.1 ± 40.4	<0.001
Carbohydrate (g/day)	261.0 ± 106.1	244.4 ± 98.7	246.2 ± 93.0	266.4 ± 98.8	<0.001
Vitamin A (μgRE)	377.4 ± 520.5	408.1 ± 557.2	426.9 ± 536.8	432.5 ± 597.7	<0.001
Vitamin C (mg/day)	62.6 ± 69.9	63.0 ± 74.3	65.3 ± 72.3	77.9 ± 151.5	<0.001
Calcium (mg/day)	321.4 ± 180.2	347.4 ± 180.4	381.1 ± 202.5	427.7 ± 219.4	<0.001
Sodium (mg/day)	2943.9 ± 2108.6	3510.4 ± 2266.9	4057.1 ± 2657.8	4315.5 ± 2796.5	<0.01

Values are given as the number of subjects, the percentage for categorical variables, and mean ± SD for continuous variables.

**Table 2:** Associations of UPF consumption with overweight or obesity.

	Quartile of UPFs (g/day)				
	Q1	Q2	Q3	Q4	*p* Trend
Overweight or obesity					
Median	3.3	10.9	23.5	60.8	
Model 1	1.00 (ref)	1.16 (1.10, 1.23) ***	1.14 (1.08, 1.21) ***	1.08 (1.02, 1.14) *	0.182
Model 2	1.00 (ref)	1.14 (1.08, 1.21) ***	1.13 (1.06, 1.20) ***	1.08 (1.02, 1.14) *	0.690
Model 3	1.00 (ref)	1.14 (1.07, 1.20) ***	1.10 (1.04, 1.17) **	1.03 (0.97, 1.09)	0.241
Overweight					
Median	3.3	11.6	26.5	67.0	
Model 1	1.00 (ref)	1.18 (1.10, 1.25) ***	1.13 (1.06, 1.21) ***	1.08 (1.01, 1.15) *	0.929
Model 2	1.00 (ref)	1.15 (1.08, 1.22) ***	1.11 (1.04, 1.19) ***	1.07 (1.01, 1.15) *	0.836
Model 3	1.00 (ref)	1.15 (1.08, 1.22) ***	1.10 (1.03, 1.17) ***	1.03 (0.97, 1.10)	0.236
Obesity					
Median	3.4	11.4	25.0	63.5	
Model 1	1.00 (ref)	1.11 (1.01, 1.23) *	1.19 (1.08, 1.32) ***	1.07 (0.97, 1.19)	0.739
Model 2	1.00 (ref)	1.10 (1.00, 1.22)	1.19 (1.08, 1.32) ***	1.09 (0.98, 1.20)	0.499
Model 3	1.00 (ref)	1.09 (0.99, 1.21)	1.15 (1.04, 1.27) **	1.02 (0.92, 1.14)	0.556

* *p* < 0.05, ** *p* < 0.01, *** *p* < 0.001. Model 1 adjusted gender, age, education level, individual annual income, and occupation; Model 2 further adjusted drinking past month, labor intensity, and place and region of residence based on Model 1; Model 3 further adjusted total energy, protein, fat, carbohydrate, vitamin A, vitamin C, calcium, and sodium intake based on Model 2.

**Table 3:** Stratified analyses of overweight or obesity risk and UPF consumption.

	Quartile of UPFs (g/day)				
	Q1 (<4.6)	Q2 (4.6–18.2)	Q3 (18.2–59.1)	Q4 (>59.1)	*p* for Interaction
Gender					0.006
Men	1.00 (ref)	1.18 (1.08, 1.29) ***	1.18 (1.08, 1.29) ***	1.08 (0.99, 1.17)	
Women	1.00 (ref)	1.14 (1.05, 1.23) **	1.12 (1.03, 1.22) **	1.10 (1.01, 1.20) *	
Age					0.392
18–44	1.00 (ref)	1.08 (0.98, 1.19)	1.09 (0.99, 1.20)	1.02 (0.93, 1.13)	
45–59	1.00 (ref)	1.14 (1.04, 1.25) **	1.12 (1.02, 1.23) **	1.06 (0.96, 1.17)	
≥60	1.00 (ref)	1.22 (1.07, 1.38) **	1.16 (1.02, 1.33) **	1.08 (0.93, 1.24)	
Education level					0.850
Primary school or below	1.00 (ref)	1.18 (1.05, 1.33) **	1.14 (1.01, 1.29) *	1.11 (0.97, 1.27)	
Secondary school	1.00 (ref)	1.10 (1.02, 1.19) **	1.11 (1.02, 1.20) **	1.02 (0.94, 1.11)	
College or above	1.00 (ref)	1.19 (1.03, 1.37) *	1.16 (1.00, 1.33) *	1.10 (0.96, 1.26)	
Individual annual income (CNY)					
<10,000	1.00 (ref)	1.22 (1.10, 1.37) ***	1.15 (1.02, 1.29) *	1.08 (0.95, 1.22)	0.363
10,000–39,999	1.00 (ref)	1.12 (1.03, 1.21) **	1.13 (1.04, 1.23) **	1.04 (0.96, 1.13)	
40,000–99,999	1.00 (ref)	1.06 (0.88, 1.29)	1.01 (0.84, 1.22)	0.93 (0.77, 1.12)	
>100,000	1.00 (ref)	1.21 (0.81, 1.79)	1.06 (0.73, 1.54)	1.10 (0.74, 1.64)	
No response	1.00 (ref)	0.87 (0.65, 1.15)	0.81 (0.61, 1.08)	0.85 (0.64, 1.12)	
Occupation					0.123
Student	1.00 (ref)	1.54 (0.76, 3.12)	1.60 (0.80, 3.20)	1.11 (0.58, 2.14)	
Retired or unemployed	1.00 (ref)	1.18 (1.07, 1.30) ***	1.19 (1.07, 1.32) ***	1.07 (0.95, 1.20)	
Professionals	1.00 (ref)	1.21 (1.03, 1.43) *	1.06 (0.90, 1.25)	1.07 (0.91, 1.25)	
Business service	1.00 (ref)	1.04 (0.90, 1.21)	1.15 (0.99, 1.34)	1.09 (0.94, 1.27)	
Manual labor	1.00 (ref)	1.04 (0.92, 1.17)	1.01 (0.89, 1.13)	0.99 (0.87, 1.12)	
Others	1.00 (ref)	1.25 (1.04, 1.51) *	1.15 (0.95, 1.40)	0.96 (0.79, 1.16)	
Place of residence					0.047
Metropolis	1.00 (ref)	1.10 (0.98, 1.23)	1.02 (0.91, 1.15)	0.94 (0.84, 1.06)	
Small and medium-sized cities	1.00 (ref)	1.25 (1.12, 1.38) ***	1.12 (1.01, 1.25) **	1.13 (1.01, 1.26) **	
Rural areas	1.00 (ref)	1.04 (0.95, 1.14)	1.13 (1.02, 1.24) **	1.01 (0.91, 1.11)	

* *p* < 0.05, ** *p* < 0.01, *** *p* < 0.001.

## Data Availability

Data sharing is not applicable to this study, according to the China National Center for Food Safety Risk Assessment.
